# Exploring the Relationship Between Transitional Object Attachment and Emotion Regulation in College Students

**DOI:** 10.3390/healthcare13010039

**Published:** 2024-12-29

**Authors:** Cheng-Hung Ko, Yong-Ting Liang, Yu-Chi Liao, Hui-Fang Chen

**Affiliations:** 1 Department of Addiction and Forensic Psychiatry, Jianan Psychiatric Center, Ministry of Health and Welfare, Tainan 71742, Taiwan; puppyko@gmail.com; 2 Research Center for Humanities and Social Science, National Chung Cheng University, Chiayi 62102, Taiwan; 3 Department of Psychology, College of Medical and Health Science, Asia University, Taichung 413305, Taiwan; 109014078@live.asia.edu.tw; 4 Center for Prevention and Treatment of Internet Addiction, Asia University, Taichung 413305, Taiwan; 5 Clinical Psychology Center, Asia University Hospital, Taichung 413505, Taiwan; 6 Department of Social and Behavioural Sciences, City University of Hong Kong, Kowloon, Hong Kong

**Keywords:** transitional attachment objects, emotion regulation, heart rate variability

## Abstract

**Background/Objectives**: Transitional attachment objects, such as blankets, play a critical role in childhood by helping children manage separation anxiety and regulate emotions. Although attachment to these objects often decreases as children grow older, it may persist into adulthood and influence emotion regulation and stress responses. Their influence on emotion regulation in adulthood remains uncertain. This study investigates the relationship between object attachment and emotion regulation, with a focus on responses to stress among college students. The study objectives include examining whether emotional regulation varies based on an individual’s attachment to objects and investigating the role and significance of objects in the emotional regulation of adults with object attachment. **Methods**: Forty-five participants aged 18–22 were recruited to participate and completed the Object Attachment Security Measure (OASM) and the Emotional Regulation Questionnaire (ERQ). Participants were categorized into two groups based on their OASM scores: those with attachment objects (the OA group) and those without (the control group). The OA group was randomly assigned into two experimental groups: (1) Carry-but-cannot-touch (CBCT) and (2) Carry-and-touch (CAT). The CBCT group was not allowed to physically interact with their attachment objects during the recovery phase, whereas the CAT group could do so. Psychophysiological data, including Standard Deviation of NN Intervals (SDNN) and respiratory rate, were collected during three phases: baseline, stress, and recovery. **Results**: There were no differences in the ERQ scores between the OA and control groups, as well as between the CAT and CBCT subgroups. However, physiological indicators revealed that the CAT group exhibited higher SDNN during recovery than the CBCT group, suggesting that physical interaction with the attachment object enhanced stress regulation and promoted relaxation. **Conclusions**: While object attachment did not impact self-reported emotional regulation, it did influence physiological responses to stress, indicating that attachment objects may facilitate emotional recovery through tactile interaction. These findings highlight the potential of attachment objects as adaptive tools for stress management in young adults.

## 1. Introduction

Children form multiple attachments, including connections to inanimate objects such as blankets or soft toys [1]. Winnicott [2] has emphasized the importance of attachment to a “transitional object (TO)”, which assists children in re-establishing connections with the caregiver following a separation period. A TO comforts children and helps regulate emotions during psychological conflicts [3]. It also assists children in developing a sense of mastery and autonomy, marking the early stages of engaging in individual symbolization [4]. An attachment to a TO is a normal, universal, and essential aspect of healthy development in early childhood [5].

### 1.1. Emotion Regulation

Emotion regulation refers to the processes by which emotions are regulated [6]. Emotion regulation encompasses all processes involved in altering the duration and intensity of emotions, as well as emotion-related physiological states and behaviors [6]. Specific emotion regulation strategies can be categorized based on the timeline of the emotional response process [7]. At the broadest level, emotion regulation patterns can be categorized into two strategies: antecedent-focused and response-focused strategies, where the first refers to cognitive appraisal, while the latter involves expressive suppression [8].

Cognitive reappraisal refers to a cognitive change that entails interpreting an emotion-inducing situation in a manner that alters its emotional effect [9]. Cognitive reappraisal is a strategy of the antecedent-focused pattern that takes place before the full generation of emotional response tendencies [8]. This implies that cognitive reappraisal can thus efficiently modify the entire subsequent emotion trajectory.

Expressive suppression refers to a response modulation that involves inhibiting ongoing emotion-expressive behavior [10]. It is classified as a response-focused strategy. Expressive suppression comes relatively late in the emotion-generative process, and primarily modifies the behavioral aspect of the emotion response tendencies [8]. Suppression should thus be effective in decreasing the behavioral expression of negative emotion but might have the unintended side effect of clamping down on the expression of positive emotion [8]. Expressive suppression does not directly address and reduce the experience of negative emotions. Consequently, negative emotions may persist and accumulate without resolution [8].

Expressive suppression necessitates continuous effort from the individual to regulate emotional response tendencies as they emerge. The repeated exertion for expressive suppression may deplete cognitive resources that could otherwise be allocated toward enhancing performance in other aspects [11]. The emotion regulation strategies employed by adults are often associated with their attachment styles [12]. This study attempts to investigate the association between factors related to attachment styles and the influence on emotion regulation.

### 1.2. Transitional Objects (TOs)

A transitional object (TO), often referred to as a ‘comfort’ or ‘security’ object [3], serves as a key mechanism connecting the normative object attachment with emotional processes and regulation [13]. Functionally, TOs facilitate the child’s shift from states of ‘high arousal’ to ‘low arousal’ during periods of distress [14]. An attachment to these objects contributes to self-establishment by mirroring the child’s individual unique personality traits and also plays a crucial role in emotional development [15]. During adolescence, an attachment to a TO frequently serves as a concrete expression of self-identity and is often accompanied by significant emotional investment [16].

Although researchers often believe that the attachment to a TO would gradually fade [17,18], findings are not conclusive. Litt [19] reported that most children give up these objects as they grow older [20], but over 30% of adolescents and young adults reported using TOs [21,22]. Sherman et al. [20] investigated the effect of attachment to a TO during childhood and reported no significant behavioral differences between children with and without TOs. Further studies have indicated that attachment style and help-seeking attitudes might be associated with the continuous use of a TO [18,23]. The use of TOs is associated with secure attachment in children aged 12 to 30 months [24]. Compared to securely attached children, avoidant children tend to rely more on TOs during periods of anxiety [25] and are less able to utilize the internalized model of their primary caregiver [26]. In addition, avoidant children who have formed an attachment to an object will often use this object for comfort, rather than seeking out their mother [27]. They perceive their primary caregivers as unavailable or rejecting, and reliance on TOs may be prolonged in adolescence [22].

However, the connection between object attachment and emotional regulation in adulthood remains ambiguous, necessitating further investigation. On the one hand, the continued retention of TOs beyond childhood may be indicative of emotional vulnerability in an individual [23,28,29]. Transitional objects are indicators for identifying psychological distress such as impaired interpersonal relationships in adolescents [22,23]. This suggests that object attachment during adolescence may not positively contribute to mental health in later stages of life. For example, in looking at extreme object attachment in adulthood, people who are unable to discard items are often perceived as experiencing psychological difficulties [4]. An increased dependence on these objects may be linked to a reduction in psychological resilience [30]. Persisting object attachment beyond childhood is indicative of emotional distress and may be indicative of underlying psychopathology, such as borderline personality disorder [28,29]. These people often struggle to rely on their own internal mechanisms for self-soothing during separation. These difficulties may hinder their capacity to effectively maintain and regulate relationships with significant others, particularly after experiencing the stress of separation [18]. As a result, the utilization of TOs is linked to lower general wellbeing among adolescents [23] in college-aged students [31].

On the other hand, a prolonged attachment to a TO is not necessarily indicative of psychopathology. Instead, it may reflect challenges in managing the emotionally complex developmental transition from childhood to adolescence and adulthood [30]. Since emotions are deeply intertwined with social processes and developmental trajectories, objects may serve as a social agency [32] that is influenced or driven by our emotional experiences [15], and therefore trigger or regulate emotions [32]. In other words, a TO may play a significant role in triggering or regulating emotions [15]. For adolescents experiencing pathological disturbances, possessing a TO can facilitate the resolution of separation-related issues and contribute to the development of their sense of self and their relationships with others [33]. Moreover, adults may regulate their emotions by maintaining ownership over objects that symbolize aspects of the self, represent significant relationships, or evoke hedonic sensory experiences [34]. The tendency of adults to acquire more objects and retain possessions further highlights the strong connection between attachment to objects and the regulation of emotions [15]. The function of attachment objects evolves with age and may play a significant role in mitigating stress responses [35].

This study examined whether emotional regulation would vary in participants with or without object attachment by evaluating variations in physiological and psychological indicators. Heart rate variability (HRV) [36] was chosen as the physiological indicator of emotion regulation. Heart rate variability has served as a physiological indicator that reflects the individual’s state of emotion regulation. Acute psychological stress has been shown to markedly reduce HRV, reflecting a shift towards sympathetic nervous system activation and parasympathetic withdrawal. This alteration in autonomic regulation is a well-established indicator of acute mental stress [37]. Heart rate variability is a valuable tool for assessing stress and evaluating an individual’s stress management [38].

Investigating the use of TOs in adolescents may offer valuable indicators for their emotional states and their inclination to seek or avoid help for personal and emotional challenges. Given that TO attachment beyond childhood is influenced by attachment styles [39], it can be hypothesized that emotional regulation may vary depending on the individual with or without TO attachment.

This study is structured into two sections. The first aims to examine whether emotional regulation varies based on an individual’s attachment to objects. The second component focuses on investigating the role and significance of objects in the emotional regulation of adults with object attachments. The former compares variations in scores on the Emotion Regulation Questionnaire and associated physiological indicators (e.g., HRV) during real stressful situations based on the individual with or without object attachment. The latter examines differences in scores on the Emotion Regulation Questionnaire and physiological indicators among individuals with object attachment when exposed to real stressful situations, comparing conditions where they have physical contact with the object versus when they do not.

## 2. Materials and Methods

### 2.1. Participants

Participants were recruited from a university in central Taiwan and comprised a total of 45 individuals aged 18 to 22 years. Recruitment was facilitated using convenience sampling. The sample included individuals with an attachment to TOs, as well as a control group of individuals without such attachments.

### 2.2. Materials

#### 2.2.1. Instruments

##### University Scholastic Ability Test

This test was utilized as a stress-inducing stimulus during the psychophysiological measurement phase. Participants were instructed to complete a selection of items from the 111 Academic Year Scholastic Ability Test, including five items from the Chinese Comprehensive Ability Test [40]. Additionally, two English-to-Mandarin translation questions were included: “History repeatedly proves that wars lead to extremely terrible disasters. Avoiding conflicts and ensuring world peace should be goals pursued by all humanity”. “Keeping a pet is not just a short-term experience but a lifelong commitment to an animal. While enjoying the joy pets bring us, we must not neglect the responsibility of taking good care of them”.

##### Biofeedback Device

The ProComp Infiniti™ 5.1.1 biofeedback system [41] was used to collect participants’ physiological data, including respiration (Respiration Sensor) and heart rate (Electrocardiogram [EKG] Sensor). The Standard Deviation of NN Intervals (SDNN), used as an indicator of HRV, reflects autonomic nervous system activity. Higher SDNN values indicate greater neural regulation sensitivity, while lower values suggest reduced autonomic regulation.

#### 2.2.2. Questionnaires

##### Object Attachment Security Measure (OASM)

The OASM [42] was used to assess attachment to objects. The OASM has demonstrated satisfactory internal consistency (Cronbach’s α = 0.91), test–retest reliability (ICC = 0.793 and 0.857), and convergent validity (r = 0.447 to 0.612 with the Saving Cognitions Inventory). The original questionnaire contains 25 items and was scored on a 7-point Likert scale ranging from 1 (strongly disagree) to 7 (strongly agree). Permission to use the Chinese version was obtained from the original authors. Construct validity was conducted through confirmatory factor analysis (CFA), which identified two factors with loadings ranging from 0.578 to 0.887 and 0.591 to 0.875. In the current study, the OASM demonstrated high internal consistency, with a Cronbach’s α of 0.964.

##### Emotion Regulation Questionnaire (ERQ)

The ERQ was developed by Gross and John [8] and translated into Chinese by Li and Wu [43]. The ERS measures participants’ emotional regulation strategies. It consists of 10 items rated on a 4-point Likert scale from 1 (strongly disagree) to 4 (strongly agree). The questionnaire included two factors: cognitive reappraisal strategies and expressive suppression strategies. The questionnaire has demonstrated satisfactory internal consistency: cognitive reappraisal (Cronbach’s α = 0.78), expressive suppression (Cronbach’s α = 0.66), and criterion validity: cognitive reappraisal scores are positively correlated with optimism (r = 0.39, *p* < 0.01) and promotion scores (r = 0.31, *p* < 0.01), while expressive suppression scores are positively correlated with pessimism (r = 0.29, *p* < 0.01) and prevention scores (r = 0.26, *p* < 0.01).

### 2.3. Experimental Design and Procedure

Ethical approval for this study was obtained from the Ethics Committee of China Medical University and Hospital Research Ethics Center (CRREC-112-096). The studies were conducted in accordance with the local legislation and institutional requirements. Written informed consent for participation in this study was obtained from all participants.

This study was conducted using two separate experiments, outlined in detail below.

#### 2.3.1. Experiment 1

All participants were informed about their rights and the research purpose, including that participation in the study was voluntary and that they could withdraw from the study at any time without penalty. Participants gave their consent before participation. All of them completed the Chinese version of the OASM and the ERQ to assess their level of attachment to objects and their emotional regulation strategies. Based on their responses to the OASM, participants were initially categorized into two groups: those with transitional attachment objects (termed the object attachment group, or OA group) and those without transitional attachment objects (the control group). Psychophysiological measurements were taken using a biofeedback device to record heart rate and respiration across three phases: baseline, stress, and recovery.

#### 2.3.2. Experiment 2

Participants in the OA group were asked to bring their attachment objects to the experiment. This group was then further divided into subgroups: the Carry-but-cannot-touch group (CBCT), who could carry the object but were not allowed to touch it during the experiment, and the Carry-and-touch group (CAT), who were permitted to both carry and touch the object. Prior to the commencement of the experiment, all participants were informed that they would be assigned to either the CBCT group or the CAT group. The CBCT group was not permitted to touch their attachment object during the recovery phase, while the CAT group was allowed to do so.

Psychophysiological measurements were taken using a biofeedback device to record heart rate and respiration across three phases: baseline, stress, and recovery. In the baseline phase, participants were instructed to relax naturally. The stress phase was induced by asking participants to complete five Mandarin questions and two English translation questions from the University Scholastic Ability Test within ten minutes. During the recovery phase, participants were instructed to relax as fully as possible within five minutes.

### 2.4. Analysis

Upon completion of the experiment, data from the questionnaires, HRV, and respiration were analyzed using SPSS 22.0 software. Independent *t*-test, chi-square test, and one-way ANOVA were used for group comparisons. Additionally, a two-way repeated measures ANOVA was used to examine the effects of group differences and the three experimental phases on psychophysiological indicators.

## 3. Results

### 3.1. Demographic Comparison

An independent samples *t*-test was conducted to compare the OASM scores of the AO and the control group, and a significant difference was found (*p* < 0.001). A series of chi-square tests were employed to see whether the AO and the control groups differed in sex, age, emotional regulation scores (including the cognitive reappraisal strategies [CRS] and expressive suppression strategies [ESS]); no significant group differences were found (*p* > 0.05).

### 3.2. Comparison of Psychological Indicators Between Groups Across Different Conditions

Table 1 shows that 15 participants did not have attachment objects, while the remaining 30 participants indicated having attachment objects. These 30 participants were randomly assigned to either the CBCT or CAT groups. A one-way analysis of variance (ANOVA) was conducted to examine whether these subgroups varied in terms of the OASM, ERQ total, CRS, and ESS; the results are presented in Table 2. A significant difference was found in OASM scores. A post-hoc test showed that the control group had significantly lower average scores than the other three groups. No group differences were found in terms of the ERQ total, CRS, or ESS. Further analysis of the four subgroups indicated that, regardless of whether or not participants with object attachment (OA) brought their attachment object, they exhibited similar characteristics.

### 3.3. Comparison of Physiological Indicators Between Groups Across Different Conditions

A two-way repeated measures ANOVA was conducted to compare the SDNN and respiratory rate of the AO and the control groups during three phases. The results are summarized in Table 3. The phase effect was significant for both the SDNN (F = 11.315, *p* < 0.01) and the respiratory rate (F = 15.858, *p* < 0.01). Both the AO and the control groups showed lower SDNN and higher respiratory rates during the stress phase. The interaction effect and the group effect were not significant.

However, as shown in Table 4, the two-way repeated measures ANOVA comparing the SDNN and respiratory rate of the CBCT and CAT groups revealed that the CAT group exhibited a higher SDNN, with a significant interaction effect between group and condition (F = 5.264, *p* = 0.033). This finding indicates that physical contact with the attachment object enhances physiological regulation during the recovery phase.

By contrast, the CBCT group consistently showed higher respiratory rates across all conditions compared to the CAT group. Furthermore, a significant main effect for group was observed (F = 5.639, *p* = 0.025), indicating that the CAT group demonstrated more effective recovery during the recovery phase than the CBCT group.

These findings suggest that, for individuals with an attachment object, physical interaction with the attachment object improves stress regulation and facilitates relaxation. The tactile interaction with the attachment object appears to be a key factor in effectively alleviating anxiety in stressful situations.

## 4. Discussion

This study provides evidence that maintaining an emotional bond with an attachment object beyond childhood may result in a heightened emotional attachment to that object [15]. Individuals who have OA tend to exhibit higher scores on the Object Attachment Security Measure. However, there is no significant difference in emotion regulation abilities between adults with OA attachment and those without. This signifies that attachment to objects in adulthood has no impact on an individual’s ability to regulate emotions. This finding does not provide evidence that maintaining OA beyond childhood is linked to emotional distress or vulnerability [23,28,29]. Attachment objects from childhood may have a significant influence on emotional regulation, potentially extending into adulthood [15].

Several studies have identified childhood insecurity as a key factor contributing to the development of OA [44,45,46], and it has been suggested that these individuals may be experiencing certain psychological difficulties [4] or interpersonal conflicts [46]. Insecure attachment is considered to heighten the probability of forming OA [25], as it is also associated with maladaptive emotion regulation strategies [47,48]. According to Frost and Hartl’s cognitive-behavioral model [49], OA is associated with the use of objects as a source of security, comfort, and safety [50].

Psychodynamic and attachment theories also recognize the use of objects for comfort, suggesting that when the fundamental need for social connection is thwarted, individuals will seek to ease the ensuing anxiety and compensate for this need [51]. These compensatory substitutions reflect fundamental psychological needs that are crucial for maintaining well-being [52]. Thus, the emotion regulation of adults with OA through objects does not differ significantly from that of adults without OA.

This perspective was further validated by another finding from this study. This study highlights that when an individual with OA is accompanied by the object during a stressful situation, their respiratory rate tends to be lower compared to an individual with OA who is not accompanied by the object. Additionally, during the post-stress recovery phase, individuals with OA who are accompanied by their objects exhibit higher HRV. This implies that the presence of the attachment object plays a role in promoting emotional stability for individuals with OA. Physical interaction with an attachment object appears to have a beneficial impact on emotional self-regulation. This observation is consistent with the results of the experiment conducted by Harlow and Zimmermann [53], which demonstrated that physical contact with a soft surrogate is crucial for the emotion regulation of monkeys. This finding also supports the notion that emotion regulation may operate as an automatic process [54]. Individuals with object attachment appear to engage in emotion regulation not at a conscious level when accompanied by their TO. Additionally, the object may be perceived as a symbol imbued with social significance [55]. This recognition is intricately linked to the development of individual emotions and the process of socialization, potentially influencing emotional regulation [32]. The attachment object may play a role in influencing and modulating the emotional experiences or feelings of the individual with OA [15].

Post-childhood OA is also associated with the formation and development of an individual’s self-identity [16]. The attachment object, which represents a symbol of self, elicits pleasurable sensory experiences [30] or serves as a defense against loss [34]. Objects have been shown to alleviate negative emotions such as stress, sadness, or loneliness, a phenomenon supported by research in both developmental psychology [56] and consumer science [57]. The findings of this study indicate that OA does not diminish one’s capacity for emotion regulation.

Besides utilizing a self-report questionnaire to examine whether emotional regulation differs based on having OA or not, the results of this study were derived from the analysis of physiological responses, including HRV, observed when individuals were subjected to stress and thereafter. Heart rate variability is gaining attention as an objective indicator for assessing emotion regulation, providing a complementary alternative to conventional self-report questionnaires [36,58]. It additionally offers a physiological index for capturing real-time emotional responses and regulation efforts, which may not be entirely reflected in self-report questionnaires [59].

Acute psychological stress has been shown to considerably reduce HRV measures, suggesting an activation of the sympathetic nervous system and a concurrent withdrawal of parasympathetic activity during stress [37]. Elevated vagally mediated HRV has been linked to greater cognitive resilience, appropriate emotional regulation, and improved modulation of cortisol, cardiovascular function, and inflammatory responses during tasks involving stress [60]. Resilience is a complex, multidimensional construct encompassing physiological, psychological, and genetic factors. It exhibits both inter-individual variability and intra-individual heterogeneity, which were dependent on specific domains and time periods. This construct plays a crucial role in shaping mental health, particularly in relation to emotional stability [61]. This study supports that for individuals with OA, the presence of the attachment object plays a role in enhancing resilience when confronted with stress.

In addition to demonstrating that emotional-regulation ability remains unaffected for individuals with or without OA, this study further highlights that there is no significant difference in the emotion-regulation strategies employed by individuals, regardless of whether they are attached to objects or not. Aldao and colleagues regard reappraisal as an adaptive emotion regulation strategy, whereas suppression is considered to be positively associated with maladaptation in psychopathology [62,63].

However, classifying strategies as solely ‘maladaptive’ or ‘adaptive’ can be overly simplistic, as the effectiveness of any given strategy may vary depending on the specific context, individual, and goal [64]. Emotion regulation strategies should adapt according to situational contexts or specific goal demands, reflecting the flexibility in emotional regulation [65,66]. Emotional regulation in identical situations may involve the use of varying strategies, such as maintaining calm while speaking or attempting to conceal nervousness during speech [67].

The selection and flexibility of emotion regulation strategies may be influenced not only by individual preferences for certain regulatory approaches but also by individual variations in the capacity to adaptively respond to changing situational demands, which are thought to be closely linked to an individual’s cognitive control capacities [68]. Currently, there is no available literature suggesting that an individual’s cognitive control abilities or attentional capacities vary based on the presence or absence of OA. Nevertheless, some studies have proposed that individuals with insecure attachments (avoidant attachment) may utilize cognitive control mechanisms to avoid confronting negative emotions associated with attachment experiences [69,70]. Therefore, further research is required to investigate the potential reasons why emotional regulation strategies do not vary based on whether an individual has OA or not.

Although this study suggests that the use of attachment objects may serve as a healthy and effective stress-relief mechanism and an individual’s adaptation to their environment in cases of OA, the present findings are constrained by insufficient control over potential confounding variables.

First, this study could not further investigate participants with OA who declined to bring objects into the study environment. Due to the research design’s reliance on participants’ willingness to carry objects, direct comparisons between those who opted to bring objects and those who chose not to were not possible. This study lacks information regarding how participants who chose not to carry objects respond to stress or cope with post-stress situations. It remains possible that the stress responses of the individuals described above may differ from those of the two participant groups included in this study.

Second, this study is constrained by the fact that the majority of the participants were female adults. Gender plays a significant role in OA [18,71], and it remains uncertain whether the findings of this study vary across different genders.

Third, although the measures employed allowed for data collection from a relatively large sample, relying on questionnaires and evaluating physiological responses at a single point in time in a cross-sectional design risks common error variance [72]. In addition, due to a small sample size, future research could employ a mixed methods design with a large sample size to further validate and test the veracity of the findings from this study.

## 5. Conclusions

This study reveals no substantial differences in emotion regulation between individuals with or without OA. The objects also appear to assist individuals with OA in coping with stressful situations. The TO may facilitate emotion regulation [13] and support self-development [15,16]. The present study will potentially contribute to the design of educational programs aimed at enhancing emotional regulation [73] or fostering self-efficacy [74] in childhood. This study confirmed the aforementioned findings by analyzing participants’ physiological responses during real-life stressful situations. In addition, attachment style may influence the manner in which attachment objects are utilized or discontinued. The relationship between attachment styles and emotional regulation strategies is likely to exhibit greater complexity. This perspective aligns with current understanding in the fields of developmental psychology and consumer science, emphasizing the need for a more nuanced exploration of the role of objects in the context of OA in future research.

## Figures and Tables

**Table 1:** Demographics and behavioral comparison between the OA and control groups.

	Control Group M (SD) (N = 15)	OA Group M (SD) (N = 30)	t/χ^2^ *p*	Cohen’s d
Sex (M/F)	3/12	2/28	0.180	
Age (year)	19.00 (1.25)	19.50 (1.31)	0.227	-
OASM total	64.40 (27.57)	106.73 (22.90)	<0.001 *	1.727
ERQ total	24.40 (3.5)	23.23 (2.90)	0.242	-
CRS	18.53 (2.45)	17.37 (1.77)	0.074	-
ESS	5.87 (2.61)	5.87 (2.46)	1.000	-

OA: objective attachment; M/F: male/female; OASM: Object Attachment Security Measure; ERQ: Emotion Regulation Questionnaire; CRS: cognitive reappraisal strategies; ESS: expressive suppression strategies. * *p* < 0.001.

**Table 2:** Repeated measures ANOVA between groups and physiological indicators.

		Control Group M (SD) (N = 15)	OA Group M (SD) (N = 30)	Interaction Effect F	Group Effect F	Phase Effect F
SDNN	Baseline	48.71 (18.53)	47.00 (16.92)	0.276	0.340	6.164 **
	Stress	46.71 (13.03)	43.00 (13.19)			
	Recovery	55.43 (25.95)	52.45 (16.84)			
Respiratory rate (per minute)	Baseline	13.09 (2.69)	13.41 (2.63)	0.604	0.804	8.359 ***
	Stress	14.68 (1.53)	14.86 (1.46)			
	Recovery	13.52 (2.34)	14.46 (1.97)			

OA: objective attachment; SDNN: Standard Deviation of NN Intervals; ** *p* < 0.01, *** *p* < 0.001.

**Table 3:** Demographics and behavioral comparison between CBCT and CAT.

	CBCT (N = 15) M (SD)	CAT (N = 15) M (SD)	t/χ^2^ *p*	Cohen’s d
Sex (M/F)	1/14	1/14	1.000	
Age (year)	19.33 (1.29)	19.67 (1.35)	0.494	-
OASM total	105.93 (21.49)	107.53 (24.96)	0.852	-
ERQ total	23.13 (2.45)	23.33 (3.37)	0.854	-
CRS	17.13 (1.6)	17.6 (1.96)	0.480	-
ESS	6 (2.24)	5.73 (2.74)	0.772	-

OASM: Object Attachment Security Measure; ERQ: Emotion Regulation Questionnaire; CRS: cognitive reappraisal strategies; ESS: expressive suppression strategies; CBCT: Carry-but-cannot-touch group; CAT: Carry-and-touch group (CAT).

**Table 4:** Repeated measures ANOVA between CBCT and CAT groups.

		CBCT (N = 15) M (SD)	CAT (N = 15) M (SD)	Interaction Effect F	Group Effect F	Phase Effect F
SDNN	Baseline	41.91 (11.50)	52.08 (20.30)	5.264 *	2.597	15.273 **
	Stress	40.14 (8.55)	44.46 (19.04)			
	Recovery	44.31 (9.97)	59.66 (19.28)			
Respiratory rate (per minute)	Baseline	14.43 (2.99)	12.39 (1.79)	1.874	5.639 *	6.388 **
	Stress	15.18 (1.38)	14.53 (1.50)			
	Recovery	15.09 (2.15)	13.83 (1.61)			

OA: objective attachment; SDNN: Standard Deviation of NN Intervals; * *p* < 0.05, ** *p* < 0.01.

## Data Availability

Data are not publicly available due to privacy and ethical restrictions but are available from the corresponding author (Y.-C.L.) upon reasonable request.
